# Identification of genes associated with dissociation of cognitive performance and neuropathological burden: Multistep analysis of genetic, epigenetic, and transcriptional data

**DOI:** 10.1371/journal.pmed.1002287

**Published:** 2017-04-25

**Authors:** Charles C. White, Hyun-Sik Yang, Lei Yu, Lori B. Chibnik, Robert J. Dawe, Jingyun Yang, Hans-Ulrich Klein, Daniel Felsky, Alfredo Ramos-Miguel, Konstantinos Arfanakis, William G. Honer, Reisa A. Sperling, Julie A. Schneider, David A. Bennett, Philip L. De Jager

**Affiliations:** 1 Program in Translational NeuroPsychiatric Genomics, Institute for the Neurosciences, Departments of Neurology and Psychiatry, Brigham and Women’s Hospital, Boston, Massachusetts, United States of America; 2 Program in Medical and Population Genetics, Broad Institute, Cambridge, Massachusetts, United States of America; 3 Center for Alzheimer Research and Treatment, Department of Neurology, Brigham and Women’s Hospital, Boston, Massachusetts, United States of America; 4 Harvard Medical School, Boston, Massachusetts, United States of America; 5 Rush Alzheimer’s Disease Center, Rush University Medical Center, Chicago, Illinois, United States of America; 6 Department of Neurological Sciences, Rush University Medical Center, Chicago, Illinois, United States of America; 7 Harvard T.H. Chan School of Public Health, Boston, Massachusetts, United States of America; 8 Department of Diagnostic Radiology and Nuclear Medicine, Rush University Medical Center, Chicago, Illinois, United States of America; 9 Department of Psychiatry, University of British Columbia, Vancouver, British Columbia, Canada; 10 Department of Biomedical Engineering, Illinois Institute of Technology, Chicago, Illinois, United States of America; 11 Center for Translational & Systems Neuroimmunology, Department of Neurology, Columbia University Medical Center, New York, New York, United States of America; University of California San Francisco Memory and Aging Center, UNITED STATES

## Abstract

**Introduction:**

The molecular underpinnings of the dissociation of cognitive performance and neuropathological burden are poorly understood, and there are currently no known genetic or epigenetic determinants of the dissociation.

**Methods and findings:**

“Residual cognition” was quantified by regressing out the effects of cerebral pathologies and demographic characteristics on global cognitive performance proximate to death. To identify genes influencing residual cognition, we leveraged neuropathological, genetic, epigenetic, and transcriptional data available for deceased participants of the Religious Orders Study (*n =* 492) and the Rush Memory and Aging Project (*n =* 487). Given that our sample size was underpowered to detect genome-wide significance, we applied a multistep approach to identify genes influencing residual cognition, based on our prior observation that independent genetic and epigenetic risk factors can converge on the same locus. In the first step (*n =* 979), we performed a genome-wide association study with a predefined suggestive *p <* 10^−5^, and nine independent loci met this threshold in eight distinct chromosomal regions. Three of the six genes within 100 kb of the lead SNP are expressed in the dorsolateral prefrontal cortex (DLPFC): *UNC5C*, *ENC1*, and *TMEM106B*. In the second step, in the subset of participants with DLPFC DNA methylation data (*n =* 648), we found that residual cognition was related to differential DNA methylation of *UNC5C* and *ENC1* (false discovery rate < 0.05). In the third step, in the subset of participants with DLPFC RNA sequencing data (*n =* 469), brain transcription levels of *UNC5C* and *ENC1* were evaluated for their association with residual cognition: RNA levels of both *UNC5C* (estimated effect = −0.40, 95% CI −0.69 to −0.10, *p =* 0.0089) and *ENC1* (estimated effect = 0.0064, 95% CI 0.0033 to 0.0096, *p =* 5.7 × 10^−5^) were associated with residual cognition. In secondary analyses, we explored the mechanism of these associations and found that *ENC1* may be related to the previously documented effect of depression on cognitive decline, while *UNC5C* may alter the composition of presynaptic terminals. Of note, the *TMEM106B* allele identified in the first step as being associated with better residual cognition is in strong linkage disequilibrium with rs1990622^A^ (*r*^2^ = 0.66), a previously identified protective allele for TDP-43 proteinopathy. Limitations include the small sample size for the genetic analysis, which was underpowered to detect genome-wide significance, the evaluation being limited to a single cortical region for epigenetic and transcriptomic data, and the use of categorical measures for certain non-amyloid-plaque, non-neurofibrillary-tangle neuropathologies.

**Conclusions:**

Through a multistep analysis of cognitive, neuropathological, genomic, epigenomic, and transcriptomic data, we identified *ENC1* and *UNC5C* as genes with convergent genetic, epigenetic, and transcriptomic evidence supporting a potential role in the dissociation of cognition and neuropathology in an aging population, and we expanded our understanding of the *TMEM106B* haplotype that is protective against TDP-43 proteinopathy.

## Abstract 2

Philip De Jager and colleagues conduct a multistep analysiso of genetic, epigenetic and transcriptional data to identify genes associated with residual cognition.

## Author summary

**Why was this study done?:**

- Only a part of cognitive impairment in older adults is explained by common neuropathologies such as amyloid plaques, neurofibrillary tangles, stroke, or Lewy bodies found in Parkinson disease and some forms of dementia.
- Understanding the molecular basis of dissociation between cognitive outcomes and neuropathological burden is of critical importance in understanding prognosis and in designing clinical trials to prevent dementia.
- Animal studies cannot fully address this complex discrepancy between cognition and neuropathology, and a well-powered large-scale genome-wide association study with detailed cognitive and pathological phenotypes is not yet available.
- The availability of genetic, epigenetic, and transcriptomic data from two longitudinal cohorts with quantitative cognitive and pathological assessments enabled us to look for convergence of different molecular features onto certain target genes.

**What did the researchers do and find?:**

- In two well-characterized cohorts of aging (the Religious Orders Study and the Rush Memory and Aging Project), we first captured the gap between cognitive performance proximate to death and neuropathological burden for each individual (“residual cognition”).
- Then, we performed sequential analyses including a genome-wide association study (step 1), in which we identified loci that were further analyzed for association of their methylation pattern (step 2) and brain RNA expression (step 3) with residual cognition.
- We identified two genes (*UNC5C* and *ENC1*) with converging evidence from all three analytic steps, and also showed that a previously reported risk gene for frontotemporal lobar degeneration, *TMEM106B*, might play a role in the dissociation of cognition and neuropathology in older adults. Further, *UNC5C* brain expression level was correlated with presynaptic protein levels, while *ENC1* brain expression level was correlated with depressive symptoms.

**What do these findings mean?:**

- *ENC1*, *UNC5C*, and *TMEM106B* had converging evidence suggesting that they might have a role in determining cognitive resilience in the aging population, which is affected by Alzheimer disease, stroke, and other neuropathologies.
- These genes should be studied further to expand our understanding of molecular mechanisms relevant to cognitive resilience that could be translated into prognostic and therapeutic tools for dementia prevention.

## Introduction

It is well known that cognitive impairment in older adults is only partially explained by common neuropathologies such as Alzheimer disease (AD), stroke, and Lewy body disease [1–4]. Previous studies have shown that the majority of the variability in cognitive decline is unexplained, even when quantitative indices of common neuropathologies and demographic variables are included in the model [1,2]. Although additional variability has been explained by traits such as depressive symptoms [2,5] and by neuroimaging measures of brain tissue integrity [6,7], a large part of the cognitive impairment in late life remains unexplained. Further disentangling this dissociation of pathological burden and cognitive outcome is of critical importance in understanding clinical heterogeneity in the patient population and in designing treatments that can prevent progression into dementia.

To account for this dissociation, multiple mechanisms have been suggested: brain reserve capacity [2,8,9], cognitive reserve [2,10], differential neuroglial reactions to pathology [11], and other undetected pathophysiological processes [11–13]. Although prior studies have shown that the synaptic density and brain expression levels of multiple proteins in various biochemical pathways are correlated with resilient cognition [14,15], the molecular and genetic underpinnings of the dissociation of cognition and pathology remain poorly understood, and currently there are no known genetic or epigenetic determinants of the cognition–pathology discordance [16].

To identify genes that contribute to differential cognitive outcomes in the setting of neuropathology, we leveraged data from two large and richly phenotyped longitudinal cohort studies, the Religious Orders Study (ROS) and the Rush Memory and Aging Project (MAP) [17,18]. Similar to prior studies using data from ROS and MAP [1,2,4], we quantified the dissociation of cognition proximate to death and neuropathology by capturing the residual of global cognitive performance proximate to death after regressing out the effects of demographic characteristics and neuropathologies. We use the term “residual cognition” for this quantified residual of global cognitive performance that captures the dissociation of cognition and neuropathology.

We recently reported that both genetic and epigenetic variation can be independently associated with neuritic plaque pathology in the same chromosomal locus: for example, we see such convergence of molecular evidence in *ATP binding cassette subfamily A member 7 (ABCA7)* and *bridging integrator 1 (BIN1)* [19]. In this study, we designed a strategy involving a multistep analysis to identify loci in which genetic and epigenetic variation converge to influence residual cognition. In the first step of the analysis, we performed a genome-wide association study (GWAS) and identified independent loci meeting our predetermined threshold of suggestive genetic association with residual cognition. Candidate genes were identified based on proximity to the lead single nucleotide polymorphism (SNPs) from each locus and were selected for further analysis based on expression in human frontal cortex (the region for which we have additional epigenomic and transcriptomic data). For the second step, we examined associations between residual cognition and differential DNA methylation of the candidate genes in human frontal cortex. In the third step, we further validated the role of the selected genes using transcriptional data from the same cortical region. This allowed us to identify genes with converging evidence from genetic, epigenetic, and transcriptomic data regarding their roles in determining the dissociation of cognition and neuropathology.

## Methods

### Participants

This study and the protocol for both ROS and MAP were approved by the institutional review board of Rush University Medical Center, and each participant signed a written informed consent and an Anatomical Gift Act document. Our participants came from two longitudinal cohort studies of older persons, ROS and MAP. ROS, started in 1994, is a longitudinal cohort study that enrolls Catholic nuns, priests, and brothers from more than 40 communities across the United States. MAP, launched in 1997, is a longitudinal cohort study that enrolls participants with diverse backgrounds and socioeconomic status from continuous care retirement communities throughout northeastern Illinois, as well as from individual homes across the Chicago metropolitan area. Participants from both cohorts were free of known dementia at the time of enrollment, and these two cohorts were designed and are managed by the same team of investigators, who capture the same cognitive measures and conduct a structured, quantitative neuropathological examination at a single site. Thus, the two cohorts were designed to be used in combined analyses. At the time of our analyses in September 2014, 1,240 participants had been enrolled in ROS, and 1,752 participants had been enrolled in MAP; 674 and 723 participants from each study were deceased, respectively. The combined follow-up rate of these studies was 97%, and the brain autopsy rate among deceased participants was 86%. Among the deceased participants, 492 ROS participants and 487 MAP participants were of European ancestry and had the complete neuropathological evaluation, cognitive testing measures, and quality-controlled genotyping data required for our step 1 analysis (*n =* 979). Further details about the ROS and MAP cohorts can be found in previous publications [17,18] and through the Rush Alzheimer’s Disease Center Research Resource Sharing Hub (https://www.radc.rush.edu/home.htm).

### Cognitive and neuropsychiatric phenotypes

Cognitive function was annually assessed in both the ROS and MAP cohorts via 19 cognitive tests, of which 17 were distributed across five cognitive domains: episodic memory, semantic memory, working memory, perceptual speed, and visuospatial ability (S2 Table). Annual measures of global cognitive performance were derived by averaging the *z-*scores from each of these 17 annual tests [17,18,20]. We used global cognition proximate to death to derive residual cognition. Separately, the residual slope of global cognitive change and the residual slopes of cognitive change in the five cognitive domains were derived through general linear mixed models, controlling for age at enrollment, sex, and education, as previously described [21]. Diagnosis of AD dementia was made by a neurologist blinded to all postmortem data, after reviewing all available clinical data at the time of death. Selected cases were also reviewed through case conferences [13,17,18]. For most of the participants (*n =* 977), depressive symptoms were measured annually with a ten-item form of the Center for Epidemiologic Studies Depression Scale (CES-D) [5]. Given the previously reported relationship between depression and residual cognitive decline [2], this measure of depressive symptoms proximate to death was used to explore the association of depression with genes implicated in residual cognition.

### Genotyping data acquisition

Genotyping was performed on either the Affymetrix GeneChip 6.0 platform (1,878 participants, 909,600 SNPs) or the Illumina OmniQuad Express platform (456 participants, 730,525 SNPs). DNA was extracted from whole blood, lymphocytes, or frozen brain tissue, as previously described [22]. To minimize population admixture, only self-declared non-Latino individuals of European ancestry were genotyped. Then, genotyping data from both platforms were processed using PLINK software, version 1.08p [23], with standard quality control (QC) metrics such as genotype success rate > 0.95, Hardy–Weinberg equilibrium *p* > 0.001, and misshap test < 1 × 10^−9^, as previously described [22,24,25]. EIGENSTRAT was used with default settings to remove population outliers and to generate a genotype covariance matrix [26], and closely related participants were removed. After these QC steps, 1,709 individuals and 750,173 autosomal markers from the Affymetrix GeneChip 6.0 platform, and 382 individuals and 627,742 autosomal markers from the Illumina OmniQuad Express platform were used for imputation. Dosages for SNPs (>35 million) were imputed on the 1000 Genomes reference (1000 Genomes Project interim phase 1 haplotypes, 2010–2011 data freeze), using BEAGLE software, version 3.3.2 [27]. All GWAS analyses in ROS and MAP filtered SNPs based on minor allele frequency (MAF) > 0.01 and imputation INFO score > 0.3, leaving about 7 million SNPs that were analyzed. Of note, each individual included in our analysis had non-missing genotype dosages of these quality-controlled markers, as any missing markers were imputed. In the current study limited to 979 participants, 859 participants were genotyped through the Affymetrix platform, and 120 participants were genotyped through the Illumina platform. In addition, *APOE* genotyping was done through a separate sequencing procedure, as previously described [17,18], and the resulting allele counts were used in our study.

### Pathological phenotypes

The derivation of pathological variables has been previously described in detail [17,18]. Briefly, each brain was inspected for ten common pathologies relating to loss of cognition in aging populations: neurofibrillary tangles, neuritic plaques, diffuse plaques, Lewy bodies, macroscopic infarcts, microscopic infarcts, atherosclerosis, arteriolosclerosis, cerebral amyloid angiopathy (CAA), and hippocampal sclerosis [13,28–33]. More specifically, neurofibrillary tangles, neuritic plaques, and diffuse plaques were counted and scaled in five brain regions: mid-frontal, temporal, inferior parietal, entorhinal cortex, and hippocampus CA1. Composite scores for each of the three pathology types were derived by scaling the counts within each of the five regions, and then taking the square root of the average of the regional scaled values to account for their positively skewed distribution [17,18,21]. CAA was graded on a five-level scale (0 to 4) in four neocortical regions (mid-frontal, angular gyrus, inferior temporal gyrus, and calcarine cortex) and averaged to derive a CAA score, as previously described [34]. Chronic macroscopic and microscopic infarcts were each dichotomized as present or absent. Atherosclerosis was scored on a four-level severity scale, and arteriolosclerosis was measured on a four-level scale by small vessel pathologies in anterior basal ganglia [35]. Nigral, limbic, and neocortical Lewy bodies were dichotomized as present or absent, as identified using immunohistochemistry. Hippocampal sclerosis was recorded as either present or absent as evaluated with H&E stain. Pathological diagnosis of AD was given for cases with high or intermediate likelihood of AD per the modified National Institute of Aging–Reagan Institute criteria [36]. For a subset of participants (*n =* 826), transactive response DNA-binding protein 43 kDa (TDP-43) proteinopathy was measured and categorized into four steps of severity as previously described [37]: no inclusions (stage 0), inclusions in amygdala only (stage 1), inclusions in amygdala as well as entorhinal cortex and/or hippocampus CA1 (stage 2), and inclusions in amygdala, neocortex, and entorhinal cortex and/or hippocampus CA1 (stage 3). In addition, a semi-quantitative six-point scale for the severity of the TDP-43 cytoplasmic inclusions was rated as previously described (*n* = 812) [38].

### DNA methylation and RNA sequencing data acquisition

In ROS and MAP, dorsolateral prefrontal cortex (DLPFC) was selected for initial multi-omics data generation, as it is relevant to multiple common neuropathologies and cognitive phenotypes in the aging population [22]. DNA methylation levels from the gray matter of DLPFC were measured using the Illumina HumanMethylation450 BeadChip, and the measurements underwent QC processing as previously described (e.g., detection *p <* 0.01 for all samples) [19,22], yielding 708 participants with 415,848 discrete CpG dinucleotide sites with methylation measurement. Any missing methylation levels from any of quality-controlled CpG dinucleotide sites were imputed using a *k*-nearest neighbor algorithm for *k* = 100 [19]. A subset of 648 participants in our study had quality-controlled genome-wide methylation data.

RNA was extracted from the gray matter of DLPFC, and next-generation RNA sequencing (RNA-Seq) was done on the Illumina HiSeq for samples with an RNA integrity score > 5 and a quantity threshold > 5 ug, as previously described [22,39]. We quantile-normalized the fragments per kilobase of transcript per million fragments mapped (FPKM), correcting for batch effect with Combat [39,40]. These adjusted FPKM values were used for analysis. A subset of 469 participants in our study had quality-controlled RNA-Seq data, and all of them had non-missing values for the expression levels of the six genes identified in step 1.

### Brain MRI data acquisition and processing

Ex vivo brain MRI data were available in a subset of 419 participants in our study. In secondary analyses exploring phenotypic correlates of identified genes, we used a composite measure of transverse relaxation rate (*R*_2_) that was previously shown to correlate with the residual slope of global cognitive decline in ROS and MAP participants [6,7]. Data acquisition and processing have been described in detail previously [7]. In brief, for each participant, each voxel’s *R*_2_ was quantified from the spin echo images to generate an *R*_2_ map. Voxelwise linear regression was run with slope of cognitive decline as a dependent variable, *R*_2_ as an independent variable, and neuropathological indices (AD, cerebrovascular disease, and Lewy body disease) and demographics (age, sex, and education) as covariates. After correcting for multiple testing, contiguous clusters of voxels associated with the slope of global cognitive decline were identified, and mean *R*_2_ values of the voxels in each cluster were calculated, which were averaged to generate a composite *R*_2_ measure.

### Presynaptic protein data acquisition and processing

For a subset of the study participants, presynaptic protein quantification (*n =* 315) and quantitative protein–protein interaction (*n =* 295) assays were done from the gray matter of three brain regions (hippocampus, mid-frontal cortex, and inferior temporal cortex), and an overall standardized score was generated as previously described [15]. Among the measurements, we selected Complexin-I, Complexin-II, and Syntaxin/SNAP-25 interaction for our secondary analyses to explore phenotypic correlations with identified genes, given their previously reported strong association with global cognitive function independent from pathological burden [15]. For a secondary evaluation of the specificity of findings to inhibitory or excitatory terminals, we also examined Munc18-1 long (M18L, GABAergic terminals) and short (M18S, GABAergic and glutamatergic terminals) isoforms as previously described (*n =* 280) [41].

### Statistical analysis

We defined “residual cognition” as the residual of global cognition proximate to death resulting from a multivariate linear model adjusting for demographic characteristics (age at death, sex, education, study cohort) and ten common neuropathologies implicated in cognitive decline in older people (neurofibrillary tangles, neuritic plaques, diffuse plaques, Lewy bodies, macroscopic infarcts, microscopic infarcts, atherosclerosis, arteriolosclerosis, CAA, and hippocampal sclerosis) [13,28–33]. Of note, TDP-43 proteinopathy was not included in this model, as the number of participants with TDP-43 measurement was limited at the time of the analysis.

A GWAS (step 1) was performed on residual cognition, modeling residual cognition as the dependent variable, genotype as the independent variable, and the top three principal components derived from the genetic covariance matrix (EV1–3) as covariates. Using PLINK version 1.08p, a linear model assuming additive genetic effects was used, and separate analyses were performed according to genotyping platform. These results were meta-analyzed using PLINK to mitigate potential confounding effects due to the combination of platforms. We used a genome-wide significance threshold of *p <* 5 × 10^−8^ and a suggestive threshold of *p <* 10^−5^, given our modest sample size. To count the number of independent loci associated with residual cognition, we used PLINK to clump SNPs within the suggestive loci with linkage disequilibrium (LD) *r*^2^ > 0.2 as a threshold. The SNP with the most significant association with residual cognition within each independent locus was selected as the lead SNP for that locus. The lead SNPs were looked up in the HaploReg database version 4.1 [42] to identify coding variants in LD (*r*^2^ > 0.2) with each of them, and selected SNPs were interrogated with the ChromHMM core 15-state model (15-state chromatin map model) from the Roadmap Epigenomics Project’s Human Epigenome Atlas [43,44] to assess their functional implications. Each lead SNP was tested for *cis*-expressive quantitative trait locus (*cis*-eQTL) association with genes within 100 kb of the SNP, as it has been reported that the majority of *cis*-regulatory variations are found within 100 kb of transcription start site (TSS) [45]. Then, among the genes within 100 kb from each lead SNP, only those with non-zero brain expression in the majority of participants (>80%) were selected for further DLPFC DNA methylation and RNA level analyses, as differential methylation would have greater functional implications in actively transcribed genomic regions.

In order to assess differential DNA methylation of candidate genes (step 2), three steps were followed, similar to a previous study [46]. In the first step, all CpGs within 100 kb of the start and stop positions of the gene were tested with linear regression for association with residual cognition. Similar to the GWAS, residual cognition was modeled as the dependent variable, each CpG methylation level as an independent variable, and technical variables (batch and mean bisulfite conversion) as covariates. In the second step, the *p*-values of all CpGs were meta-analyzed into a single observed test statistic using Fisher’s method. Finally, to get an empirical omnibus *p*-value for a given gene, 10,000 permutations were run (permuting the outcome variable, residual cognition), and the observed test statistic was compared to these randomly generated test statistics. False discovery rate (FDR) < 0.05 was used as a threshold for statistical significance. For regions with significant association between residual cognition and differential methylation, further analyses also adjusting for the lead SNP from each region and EV1–3 were done to check whether genetic and epigenetic associations were independent.

To analyze RNA expression data (step 3), linear regressions were applied with residual cognition as the dependent variable, Combat-adjusted FPKM values as the independent variable, and technical factors as covariates (RNA integrity score, log_2_[total aligned reads], postmortem interval, and number of ribosomal bases).

We performed secondary analyses of each validated candidate gene. First, to assess whether each validated candidate genetic locus is exerting its effect through a neurodevelopmental process, the association of the lead SNP from each locus with global cognition at enrollment was assessed, controlling for demographic variables. In addition, to explore the effect of candidate genetic loci on cognitive decline, the residual slope of global cognition change (“global cognitive decline”; data available in 924 participants) and the residual slope of cognitive change in each of the five cognitive domains were tested for association with the lead SNPs from validated loci, with pathologies and EV1–3 as covariates. In addition, we tested the association of each lead SNP and RNA level with traits previously shown to be associated with residual cognition (depressive symptoms proximate to death, ex vivo brain MRI composite *R*_2_, and presynaptic protein levels), controlling for demographics. As these secondary analyses were to further characterize the selected loci that were identified through the primary analyses, a threshold for significance of *p <* 0.05 was used, except when we tested for the association of each lead SNP and RNA level with cognitive domains, pathologies, or presynaptic proteins, where we used FDR < 0.05. To further investigate the relationship among the identified *TMEM106B* SNP rs11509153^A^, TDP-43 proteinopathy, and residual cognition, we tested whether rs11509153^A^ has an independent effect on residual cognition when the analysis was controlled for TDP-43 stage or a semi-quantitative severity scale. Methylation pattern and RNA expression of *GRN*, a gene functionally downstream of *TMEM106B* [37,47], were also tested for association with residual cognition.

Of note, there were six loci that were identified in step 1 but did not have DLPFC-expressed adjacent genes and were therefore excluded from the step 2 and step 3 analyses. In an exploratory analysis, we examined their genetic association with residual cognitive decline. Moreover, all representative SNPs identified in step 1 analyses were checked for *cis*-eQTL association in various brain regions using the Genotype-Tissue Expression (GTEx) project database [48].

To obtain the variance explained of the different variable types, we followed a sequential adjusted R-squared analysis. First, we calculated the adjusted R-squared by modeling the last-visit global cognitive score versus the pathological and demographic variables. Then, to determine the variance explained by genetic, epigenetic, and transcriptomic data, we calculated the additional adjusted R-squared gained when modeling the last global cognitive score versus pathological, demographic, and genetic/epigenetic/transcriptomic data (the top SNP for each respective locus, the top respective CpGs, and RNA levels) for *UNC5C*, *ENC1*, and *TMEM106B*.

Of note, all statistical analyses were done with R 3.2.1 (https://www.r-project.org/) unless mentioned otherwise. Each analysis was limited to participants with non-missing values.

## Results

### Description of participants, data, and phenotypes

From a total of 1,397 deceased ROS and MAP participants, we included 979 deceased individuals of European descent from ROS and MAP who had complete neuropathological evaluation, cognitive testing measures, and genotyping data in our step 1 analysis. Of note, among deceased participants, included and excluded participants had similar demographic characteristics (S1 Table). Phenotypic and genetic data were measured and derived as described in the Methods and previous studies [17,18,22]. In brief, global cognition was calculated from 17 different neuropsychological tests (S2 Table), and each participant underwent a structured, quantitative neuropathological examination at the time of death. The demographic characteristics of the participants used in our analyses are reported in Table 1.

**Table 1:** Demographic characteristics of participants.

Characteristic	ROS	MAP	Combined
Cohort size, *n*	492	487	979
Age (years) at enrollment, mean (SD)	78.3 (7.0)	83.5 (5.7)	80.9 (6.9)
Age (years) at death, mean (SD)	87.8 (6.8)	89.8 (5.8)	88.8 (6.4)
Female, *n* (percent)	308 (62.6%)	322 (66.1%)	630 (64.4%)
Education (years), mean (SD)	18.2 (3.3)	14.5 (2.8)	16.4 (3.6)
Diagnosis of AD dementia, *n* (percent)	209 (42.9%)	184 (38.1%)	393 (40.5%)
Pathological diagnosis of AD, *n* (percent)	312 (63.4%)	312 (64.1%)	624 (63.7%)
Last global cognition, mean (SD)	−0.92 (1.26)	−0.90 (1.11)	−0.91 (1.19)

Among 979 participants, only 970 participants were evaluated for final diagnosis of AD dementia. Last global cognition is the measure of global cognitive performance proximate to death (*z-*score derived from baseline mean and standard deviation).

AD, Alzheimer disease; MAP, Rush Memory and Aging Project; ROS, Religious Orders Study; SD, standard deviation.

In this study, “residual cognition” was defined as the residual of global cognitive performance proximate to death, after controlling for demographic characteristics (sex, age, years of education, and study cohort) and for ten common cerebral pathologies (neurofibrillary tangles, neuritic plaques, diffuse plaques, Lewy bodies, macroscopic infarcts, microscopic infarcts, atherosclerosis, arteriolosclerosis, CAA, and hippocampal sclerosis). TDP-43 pathology was initially available for only a subset of participants and was therefore not included in the derivation of residual cognition. We note that our approach captures not only the extent to which someone might be performing better than expected but also the extent to which other individuals are performing worse than expected, based on their pathological burden.

### Genome-wide association study for residual cognition

**Fig 1:**
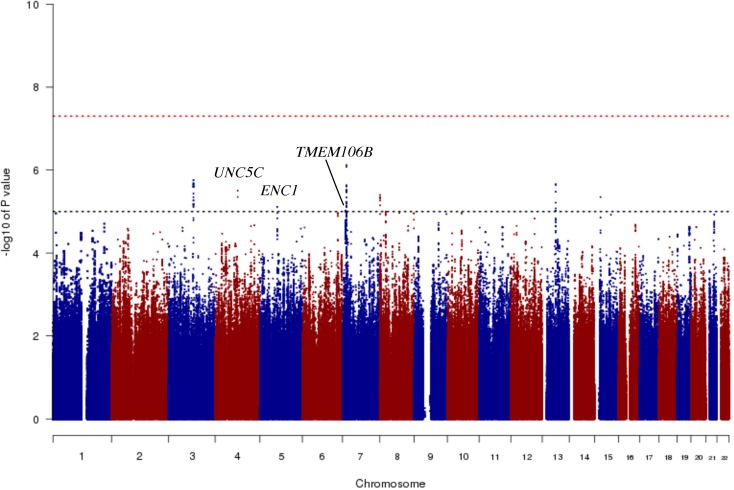
Manhattan plot from the genome-wide association study for residual cognition. In this plot, we present the results for each SNP tested in the genome-wide association study that included 979 participants from the Religious Orders Study and the Rush Memory and Aging Project. Each point is one SNP. The *x*-axis denotes the physical position of the SNP, and the *y*-axis reports −log(*p-*value) for each SNP. The threshold for a suggestive association (*p <* 10^−5^) is denoted by the black dotted line and identifies those loci that were considered in step 2 of our analysis. The red dotted line denotes the threshold of genome-wide significance. The three loci considered in step 2 are highlighted: *UNC5C*, *ENC1*, and *TMEM106B*.

**Fig 2:**
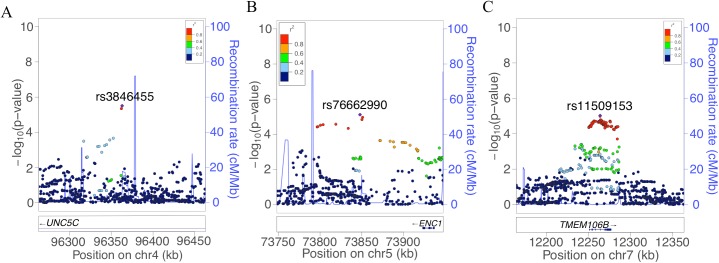
Regional genetic association plots for *UNC5C*, *ENC1*, and *TMEM106B* regions. In these regional plots, we present the association results for all SNPs (dots) within each region of interest. In each region, the lead SNP is colored in purple, and other SNPs are colored based on their extent of linkage disequilibrium with the lead SNP, following the color key included at the top right of each panel. The *x*-axis denotes the physical position of the SNP, and the *y*-axis reports −log(*p-*value) for each SNP. The blue line denotes the recombination rate in this region in EUR participants from the 1000 Genomes Project. The location of the gene is presented at the bottom of the figure. (A) *UNC5C* region where rs3836455 (hg19 chr4:96363012) is the lead SNP associated with residual cognition. (B) *ENC1* region where rs76662990 (hg19 chr5:73847916) is the lead SNP. (C) *TMEM106B* region where rs11509153 (hg19 chr7:12263800) is the lead SNP. Regional genetic association plots were plotted with LocusZoom [49].

In order to identify genetic effects on residual cognition, we performed a GWAS for this trait (Fig 1) as a first step of our analyses. The minor allele of each lead SNP was used as the alternate allele in this analysis; thus, in some cases the minor allele may be protective while in other cases the minor allele may be deleterious in relation to residual cognition. Given our moderate sample size, no variant met a threshold of genome-wide significance (*p <* 5 × 10^−8^); however, 67 variants across nine independent loci in eight distinct genomic regions met our predefined threshold for suggestive results (*p <* 10^−5^) (S3 Table). The lead SNPs with the smallest *p*-value within each independent locus are shown in Table 2. There were six genes within 100 kb of these lead SNPs: *unc-5 netrin receptor C (UNC5C)*, *ectodermal-neural cortex 1 (ENC1)*, *transmembrane protein 106B (TMEM106B)*, *anterior gradient 2 (AGR2)*, *anterior gradient 3 (AGR3)*, and *LOC286083*, as defined by the Genome Reference Consortium GRCh37.p13 primary human genome assembly. We also checked whether identifying genes based on LD patterns would change the candidate genes: five genes (*UNC5C*, *ENC1*, *TMEM106B*, *AGR3*, and *LOC286083*) included SNPs that were in LD with the lead SNPs, and all of these genes were also captured by our 100-kb cutoff. All identified SNPs were either intronic or intergenic, but a missense *TMEM106B* variant on Chromosome 7, rs3173615^G^, was in LD (*r*^2^ = 0.67) with the local lead SNP, rs11509153. Among the lead SNPs, only rs11509153^A^ (within *TMEM106B*) exhibited a *cis*-eQTL effect with respect to the genes within 100 kb in the DLPFC of ROS and MAP participants, being associated with lower expression of *TMEM106B* (estimated effect = −0.22, 95% CI −0.35 to −0.10, *p =* 6.4 × 10^−4^). Three of the six genes within 100 kb from lead SNPs had non-zero DLPFC transcript levels in a majority (>80%) of samples from ROS and MAP participants: *UNC5C* (median [first quartile–third quartile] adjusted FPKM = 1.60 [1.41–1.77]), *ENC1* (adjusted FPKM = 70.10 [45.54–94.34]), and *TMEM106B* (adjusted FPKM = 3.55 [2.95–4.25]). Therefore, these three genes could be evaluated in all three steps of our analysis. Regional genetic association plots around the lead SNPs close to these genes are depicted in Fig 2.

**Table 2:** Lead SNPs for each independent locus associated with residual cognition.

CHR	Position (hg19)	SNP	Major	Minor	MAF	*p-*Value	Estimated effect	Genes within 100 kb
3	105772040	rs60328885	G	A	0.10	1.7 × 10^−6^	−0.34	—
4	96363012	rs3846455	C	G	0.07	3.1 × 10^−6^	−0.42	*UNC5C* (0 kb)
5	73847916	rs76662990	A	G	0.11	7.7 × 10^−6^	0.30	*ENC1* (−75.32 kb)
7	12263800	rs11509153	G	A	0.41	9.4 × 10^−6^	0.19	*TMEM106B* (0 kb)
7	16944069	rs74665712	C	T	0.07	7.6 × 10^−7^	−0.58	*AGR2* (+99.33 kb), *AGR3* (+22.46 kb)
7	17065965	rs1029576	C	G	0.41	4.6 × 10^−6^	−0.20	—
8	1216767	rs34130287	G	C	0.21	4.0 × 10^−6^	−0.25	*LOC286083* (−27.53 kb)
13	57529602	rs9527561	G	A	0.46	2.2 × 10^−6^	−0.21	—
15	25772908	rs7402241	C	T	0.04	4.5 × 10^−6^	−0.50	—

Estimated effect indicates change in residual cognition, as measured by *z-*score, per each additional minor allele of each SNP.

CHR, chromosome; MAF, minor allele frequency; SNP, single nucleotide polymorphism.

By contrast, consistent with our group’s prior report showing that *APOE* genotype loses its effect on cognition when the analysis is controlled for pathology [50], *APOE* ε4 count and *APOE* ε2 count were not associated with residual cognition (FDR > 0.05). Further, none of the 19 SNPs reaching overall genome-wide significance (*p <* 5 × 10^−8^) in the International Genomics of Alzheimer’s Project analysis [51] had an association with residual cognition (FDR > 0.05). Finally, none of the loci associated with cognitive traits in large GWASs [52,53] or that were cerebral cortex expression quantitative trait loci in a recent meta-analysis [54] overlapped with the suggestive residual cognition loci from step 1.

### Epigenetic and transcriptomic studies of selected genes from the genome-wide association study

**Fig 3:**
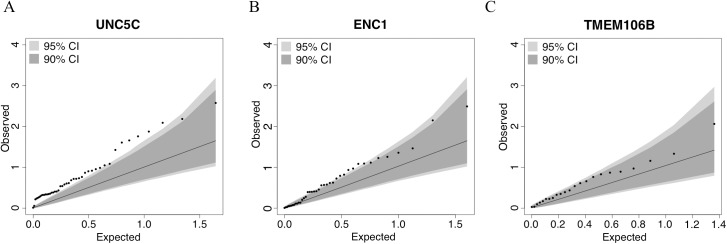
Quantile–quantile plots for the association of residual cognition and DNA methylation pattern in *UNC5C*, *ENC1*, and *TMEM106B* regions. Each panel presents the results of the DNA methylation analysis of one of the three tested regions: (A) *UNC5C*, (B) *ENC1*, and (C) *TMEM106B*. The omnibus analysis assesses the level of evidence of association for the entire region; here, we illustrate the results by plotting the association statistic for each CpG (dots) comparing the observed *p-*value to the value expected from a null distribution. The dark gray area in each plot denotes the 90% confidence interval, and the light gray area denotes the 95% confidence interval. The functional unit of methylation is not a single CpG but rather a methylated region, and we see that the *ENC1* and *UNC5C* regions demonstrate a level of association that is globally different from what one would expect by chance.

In step 2, we defined each gene’s genic region as the chromosomal segment containing the transcribed elements of each gene ±100 kb of flanking DNA. Then, in the subset of the study population that had DNA methylation data (*n =* 648), we identified CpG dinucleotides within each genic region that were detected in our DLPFC samples using the Illumina HumanMethylation450 array [19,22]. Using the set of CpGs in each genic region, we calculated an omnibus score that summarized the evidence of association between methylation levels and residual cognition in each of the three genic regions, as previously described [46]. The methylation patterns of *UNC5C* and *ENC1* regions were associated with residual cognition (FDR < 0.05; Table 3; Fig 3), and therefore these loci displayed converging evidence of genetic and epigenetic association. These associations persisted when the analyses were controlled for the respective genotypes implicated in residual cognition (lead SNP from step 1; Table 2): the methylation associations are therefore not driven by the SNPs identified in step 1. In step 3, in the subset of the study population that had DLPFC RNA-Seq data (*n =* 469), residual cognition was associated with mRNA levels of both *UNC5C* (estimated effect = −0.40, 95% CI −0.69 to −0.10, *p =* 0.0089) and *ENC1* (estimated effect = 0.0064, 95% CI 0.0033 to 0.0096, *p =* 5.7 × 10^−5^). By contrast, neither the DLPFC methylation pattern nor mRNA level of *TMEM106B* was associated with residual cognition. Thus, in our multistep analysis, two genes (*UNC5C* and *ENC1*) had converging genetic, epigenetic, and transcriptomic evidence for a role in determining residual cognition.

**Table 3:** Association of differential DNA methylation patterns with residual cognition.

Gene	Number of CpGs	OmniBus *p-*value	Omnibus FDR	Top CpG	*p*-Value	FDR	DLPFC chromatin state
*UNC5C*	44	0.0025	0.008	cg10528218	0.0027	0.117	Enhancer
*ENC1*	40	0.0271	0.041	cg18892446	0.0032	0.129	Weak transcription
*TMEM106B*	23	0.0937	0.094	cg09613507	0.0086	0.199	Weak transcription
**After adjustment for associated SNP**							
*UNC5C*	44	0.0028	NA	cg10528218	0.0015	0.066	
*ENC1*	40	0.0204	NA	cg18892446	0.0038	0.082	

DLPFC, dorsolateral prefrontal cortex; FDR, false discovery rate.

### An *UNC5C* allele, rs3846455^G^, is associated with more rapid episodic memory decline

To further characterize the two genetic regions with convergent genetic/epigenetic/transcriptomic evidence, we performed additional analyses to begin to assess whether their effect may be related to cognitive loss with advancing age or a higher cognitive attainment during development and early life. We therefore picked the minor alleles of the lead SNPs from each of the loci (Table 1): rs3846455^G^, within the first intron of *UNC5C*, and rs76662990^G^, close to *ENC1*. Both SNPs were then evaluated in greater detail in relation to predicted chromatin state, baseline cognitive scores, an individual’s pathology-adjusted slope of cognitive decline, and pathological burden. Finally, we examined each SNP’s associations with previously reported correlates of residual cognitive decline in ROS and MAP participants (depressive symptoms [2] and composite MRI *R*_2_ score [6,7]).

**Fig 4:**
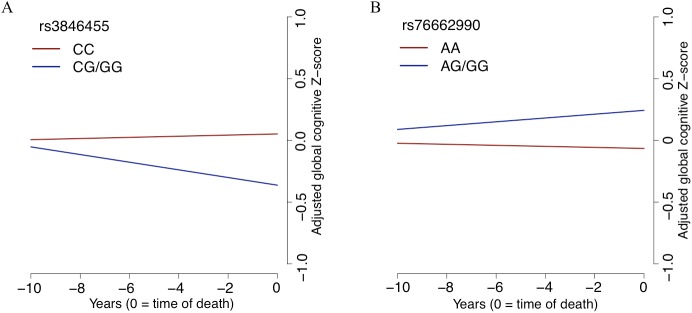
The slope of global cognitive decline by genotype. The average slope of global cognitive decline of individuals with a certain genotype is shown, after adjusting for pathology and demographics. In both panels, the *x*-axis reports years before the participant’s death, and the *y*-axis presents pathology-adjusted global cognition (*z-*score, derived from baseline mean and standard deviation). The slope is the pathology-adjusted residual slope of global cognitive decline, and the intercept represents mean residual cognition. (A) All Religious Orders Study and Rush Memory and Aging Project participants are partitioned by their genotype at rs3846455 in the *UNC5C* locus. Cognition is declining faster for participants with one or two copies of the minor allele (blue line) than for individuals who are homozygous for the major allele (red line). (B) The results for participants partitioned by rs76662990 genotype, near the *ENC1* locus. Here, the presence of the minor allele is protective against pathology-adjusted cognitive decline. Of note, we grouped participants who were homozygotes for the minor allele together with the heterozygotes in this plot, as homozygotes were rare for both SNPs.

According to the 15-state chromatin map model of human brain regions from the Roadmap Epigenomics Project’s Human Epigenome Atlas [43,44], rs3846455, which is found within the first intron of *UNC5C*, is in a region annotated as an enhancer in multiple brain regions, including the middle hippocampus, substantia nigra, cingulate gyrus, inferior temporal lobe, and angular gyrus. However, it is in a quiescent chromatin conformation in the DLPFC. The frequency of the rs3846455^G^ allele associated with lower residual cognition in our step 1 analysis (ROS and MAP MAF = 0.07) is comparable to that reported for the 1000 Genomes Project phase 1 EUR population (MAF = 0.06) [42]. When adjusted for baseline demographic variables, rs3846455^G^ dosage was not associated with a participant’s baseline global cognitive score (*p >* 0.05). However, after adjusting for pathology and demographic variables, rs3846455^G^ dosage was associated with more rapid global cognitive decline (estimated effect = −0.033, 95% CI −0.050 to −0.016, *p =* 1.9 × 10^−4^). These two analyses suggest that the detrimental effect of the *UNC5C* rs3846455^G^ allele may be related to processes in later life and is less likely to be related to developmental phenomena. The slopes of global cognitive decline for the participants with and without the minor allele (rs3846455^G^) are depicted in Fig 4. We further evaluated the rates of decline in the five cognitive domains that were included in the global cognitive score, and the detrimental effect of the *UNC5C* rs3846455^G^ allele on global cognitive decline appears to be primarily driven by more rapid episodic memory decline (estimated effect = −0.037, 95% CI −0.054 to −0.019, FDR = 2.3 × 10^−4^); other cognitive domains were not associated (FDR > 0.05) (S4 Table).

In terms of pathological measures, we tested for association with rs3846455^G^, controlling for age at death, sex, and study cohort. None of the ten pathological traits tested are associated with rs3846455^G^ dosage (FDR > 0.05), as expected given our study design. In addition, TDP-43 stage, which was not included in our initial model, was not associated with rs3846455^G^ dosage (*p >* 0.05).

For previously reported phenotypic correlates of residual cognitive decline [2,7,15], neither depressive symptoms proximate to death (*p >* 0.05) nor composite *R*_2_ measures from ex vivo brain MRI (*p >* 0.05) were associated with rs3846455^G^ dosage. Similarly, we found no association of *UNC5C* RNA level with either depressive symptoms proximate to death (*p >* 0.05) or the composite *R*_2_ (*p >* 0.05). On the other hand, rs3846455^G^ dosage was associated with the expression of certain presynaptic proteins previously reported to be associated with residual cognitive decline in the ROS and MAP cohorts [15]: rs3846455^G^ was associated with lower Complexin-I level (estimated effect = −0.33, 95% CI −0.57 to −0.09, *p =* 0.0073, FDR = 0.022) but not with Complexin-II level (FDR > 0.05) or Syntaxin/SNAP-25 protein–protein interaction (FDR > 0.05). Higher *UNC5C* RNA level was associated with lower Complexin-I level (estimated effect = −0.69, 95% CI −1.09 to −0.30, *p =* 6.1 × 10^−4^, FDR = 0.0018), lower Complexin-II level (estimated effect = −0.61, 95% CI –1.05 to −0.18, *p =* 0.0063, FDR = 0.0094), and weaker Syntaxin/SNAP-25 interaction (estimated effect = −0.45, 95% CI −0.80 to −0.09, *p =* 0.014, FDR = 0.014). Complexin-I is enriched in GABAergic, inhibitory terminals, as is the M18L isoform of Munc18-1, previously reported to be associated with cognitive decline in the MAP cohort [41]. On further exploratory analyses, we also observed an effect of rs3846455^G^ dosage on the M18L isoform (estimated effect = −0.37, 95% CI −0.70 to −0.04, *p =* 0.028) but not the M18S isoform (*p >* 0.05), supporting the association of *UNC5C* rs3846455^G^ with decreased inhibitory presynaptic terminal protein composition.

### An *ENC1* allele, rs76662990^G^, is associated with slower decline in multiple cognitive domains

In the *ENC1* locus, rs76662990^G^ is the minor allele that was associated with better residual cognition in our step 1 analysis. This SNP is found 75.32 kb downstream from the 3′ end of *ENC1*, and its MAF in ROS and MAP is 0.11, which is comparable to the 1000 Genomes Project phase 1 EUR population MAF of 0.10. rs76662990^G^ is in a quiescent chromatin state across multiple brain regions per the 15-state chromatin map model generated by the Roadmap Epigenomics Project’s Human Epigenome Atlas [43,44]. Baseline global cognition was not associated with rs76662990^G^ dosage (*p >* 0.05), after adjusting for baseline demographics. When adjusted for pathology and demographics, rs76662990^G^ dosage was modestly associated with a slower rate of adjusted global cognitive decline (additive model; estimated effect = 0.016, 95% CI 0.003 to 0.029, *p =* 0.018). The modest protective effect against adjusted cognitive decline was seen in all cognitive domains except working memory (S4 Table). Thus, the locus captured by rs76662990^G^ seems to exert its protective effect on multiple cognitive domains (S4 Table). As expected, none of the ten pathological traits tested were associated with rs76662990^G^ (FDR > 0.05). Overall, like *UNC5C*, the *ENC1* locus may be influencing processes in later life rather than developmental processes in having an effect on residual cognition.

rs76662990^G^ was not associated with either depressive symptoms proximate to death (*p >* 0.05) or the composite *R*_2_ (*p >* 0.05). On the other hand, higher *ENC1* RNA level in the DLPFC, which was associated with better residual cognition, was nominally associated with less depressive symptoms proximate to death (estimated effect = −0.0091, 95% CI −0.0165 to −0.0017, *p =* 0.016) but not with the composite *R*_2_ measure (*p >* 0.05). For presynaptic proteins, neither rs76662990^G^ dosage nor *ENC1* RNA level was associated with any of the selected measures (FDR > 0.05).

### A *TMEM106B* allele, rs11509153^A^, is associated with lower TDP-43 burden

Although the *TMEM106B* region showed no association with residual cognition in step 2, rs11509153^A^ remains of interest because it is in strong LD (*r*^2^ = 0.66) with rs1990622^G^, an allele previously reported to be associated with a lower risk of TDP-43 proteinopathy [37,55]. Thus, we looked more carefully at the *TMEM106B* locus in our secondary analyses. The rs11509153^A^ allele (ROS and MAP MAF = 0.41), which was associated with better residual cognition in step 1 (Table 1), is located in intron 4 of *TMEM106B*. In our study, rs11509153^A^ was not associated with baseline cognition (*p >* 0.05), but it was associated with slower pathology-adjusted global cognitive decline (estimated effect = 0.013, 95% CI 0.005 to 0.022, *p =* 0.0024), and this protective effect was observed in episodic memory, semantic memory, and working memory domains (FDR < 0.05) (S4 Table). Moreover, rs11509153^A^ was associated with lower *TMEM106B* RNA level in the DLPFC (*p =* 6.4 × 10^−4^), although *TMEM106B* RNA level was not associated with residual cognition (FDR > 0.05).

Since the burden of TDP-43 proteinopathy was not available in all ROS and MAP participants at the beginning of our study, we did not include this variable when calculating residual cognition. However, we completed a post hoc analysis in a reduced sample of 826 participants in which we later had this variable: we explored the possibility that the association of rs11509153^A^ with residual cognition in step 1 was due to the burden of TDP-43 proteinopathy that had not been accounted for in our measure of residual cognition. In this subset of participants with TDP-43 staging, the effect size of the association between rs11509153^A^ and residual cognition was similar to that observed in the entire study population (estimated effect = 0.18, 95% CI 0.09 to 0.28, *p =* 1.3 × 10^−4^). In line with previous reports [37,55], rs11509153^A^ was associated with lower TDP-43 stage (estimated effect = −0.16, 95% CI −0.27 to −0.05, *p =* 0.0050), and higher TDP-43 stage was associated with worse residual cognition (estimated effect = −0.090, 95% CI −0.148 to −0.032, *p =* 0.0023) in these ROS and MAP participants. Interestingly, however, even after controlling for TDP-43 stage, rs11509153^A^ remained associated with residual cognition (estimated effect = 0.17, 95% CI 0.08 to 0.26, *p =* 3.8 × 10^−4^). To examine whether a semi-quantitative measurement of TDP-43 severity better captured the burden of this pathological feature, we also tested the association of rs11509153^A^ with residual cognition while controlling for the TDP-43 severity score: the association between rs11509153^A^ and residual cognition remained similar (estimated effect = 0.18, 95% CI 0.08 to 0.27, *p =* 2.1 × 10^−4^). Thus, much of the haplotype’s protective effect was independent of the measured burden of TDP-43 proteinopathy.

We also addressed the possibility of independent effects for rs11509153^A^ and rs1990622^G^ by including both SNPs in our model: rs11509153^A^ did not have an effect beyond that captured by rs1990622^G^ (*p >* 0.05), suggesting that both SNPs are likely capturing the effect of the same functional variant. Moreover, given prior work linking the *TMEM106B* locus and the *granulin precursor (GRN)* gene, a Mendelian risk gene for frontotemporal lobar degeneration (FTLD) that is thought to be downstream of *TMEM106B* [37,47], we performed a secondary analysis of the *GRN* locus: the omnibus DNA methylation (*p =* 0.025) and mRNA level (estimated effect = −0.024, 95% CI −0.038 to −0.009, *p =* 0.0018) analyses of *GRN* revealed a modest association with residual cognition. These findings further suggest a role for *TMEM106B* and related mechanisms in influencing residual cognition in older age.

### Exploratory interrogation of the loci identified in step 1

At the conclusion of our step 1 analysis, we selected genes within 100 kb from the lead SNPs that were also expressed in DLPFC for further analyses. However, some regulatory elements can affect genes beyond 100 kb, and we may also have missed genes that are expressed in brain regions other than DLPFC. Thus, to test whether our analytic strategy might have missed pertinent candidate genes, we performed an exploratory interrogation of the lead SNPs from each region for *cis*-eQTL associations using the publically available GTEx database [48]: in multiple brain regions (anterior cingulate, frontal cortex, hippocampus, basal ganglia, cerebellum, and hypothalamus), none of the lead SNPs were *cis*-eQTLs for genes within 1 Mb from the SNP.

In addition, we explored each SNP’s association with baseline cognition and pathology-adjusted rate of global cognitive decline for the six lead SNPs from step 1 that were not further analyzed (S5 Table). Interestingly, some of the SNPs showed some association with baseline cognition (*p <* 0.05): rs60328885, rs1029576, rs9527561, and rs7402241. In particular, rs7402241^T^ displayed a highly significant association with worse baseline cognition, approaching genome-wide significance (estimated effect = −0.37, 95% CI −0.51 to −0.24, *p =* 9.0 × 10^−8^). However, this locus and the other five examined loci were not associated with cognitive traits in recent, very large GWASs of cognitive performance [52,53]. Further, rs7402241^T^ was not associated with TDP-43 or any of the ten pathologies that we used to derive residual cognition (*p >* 0.05 for all pairs). Finally, all six examined loci were associated with the pathology-adjusted slope of global cognitive decline, suggesting that they may have a role in loss of cognitive function.

### Variance in cognition proximate to death is partially explained by genetic, epigenetic, and transcriptional variations in *UNC5C*, *ENC1*, and *TMEM106B*

In a subset (*n =* 465) of participants with all genotyping, DNA methylation, and RNA-Seq data, we used a sequential adjusted R-squared analysis to obtain the variance in cognition proximate to death explained by different types of variables. In this model, demographics and common neuropathological indices explained 41.0% of the variance in global cognition proximate to death, which is consistent with prior reports from ROS and MAP [1,2]. Genetic (lead SNPs), epigenetic (lead CpGs), and transcriptomic (RNA level) variation in *UNC5C*, *ENC1*, and *TMEM106B* identified in this study explain an additional 5.9% of the variance in global cognition proximate to death. Still, more than half (53.1%) of the variance in cognition proximate to death remained unexplained.

## Discussion

Leveraging two large community-based cohorts of older adults with genetic and phenotypic data, this study used a multistep process including genetic, epigenetic, and expression analyses to identify genes associated with the dissociation of cognitive function and neuropathological burden. Our results support a potential role of *UNC5C* and *ENC1* in modulating differential neuronal susceptibility to pathological insults, and expand our understanding of *TMEM106B*, a gene known for its association with TDP-43 proteinopathy.

*UNC5C* is a netrin receptor gene that mediates repulsion from netrin signal in developmental neuronal migration and axonal guidance [56,57], and it also acts as a dependence receptor that can induce apoptosis in the absence of netrin signal [58]. A recent study linked a rare missense variant *UNC5C* T835M (rs137875858) to the risk of late-onset AD dementia and reported that *UNC5C* T835M made neurons more susceptible to neurotoxic exposures such as pathogenic β-amyloid 1–42, particularly in the hippocampus [59]. This study also reported that overexpression of *UNC5C* (T835M as well as wild-type) was associated with increased apoptosis and did not affect β-amyloid or extracellular tau production. Thus, our results that identify a convergence of genetic and epigenetic evidence within the *UNC5C* region—and that find increased *UNC5C* RNA expression to be associated with worse residual cognition—are consistent with this previous report [59]. Moreover, in our study, more rapid decline in episodic memory seemed to drive the association of the *UNC5C* allele rs3846455^G^ with cognitive decline, and this is consistent with the suggested selective effect of *UNC5C* on hippocampus [59], a brain region critical for episodic memory. Of note, *UNC5C* also has a known role in neurodevelopment; however, rs3846455^G^ was not associated with variability in cognition at study entry, making it less likely that the association between rs3846455^G^ and residual cognition is due to differential neurodevelopment. All in all, *UNC5C* is likely to be implicated in determining residual cognition through differential neuronal reaction to pathology, particularly by altering hippocampal neuronal susceptibility to pathological insults. In addition, both *UNC5C* rs3846455^G^ and higher *UNC5C* RNA level correlated with lower presynaptic protein levels, suggesting that the alterations in the synapses may be part of the functional consequences of the rs3846455^G^ risk allele and increased *UNC5C* RNA expression. Nonetheless, the functional mechanism of *UNC5C* rs3846455^G^ remains unclear: *UNC5C* rs3846455^G^ is in a quiescent chromatin state and does not influence RNA expression in the DLPFC, although the chromatin state of this locus is labeled as an enhancer in middle hippocampus according to the 15-state chromatin map model from the Roadmap Epigenomics Project’s Human Epigenome Atlas [43,44]. Also, it is unlikely that the relationship between rs3846455^G^ and residual cognition is driven by the rare *UNC5C* T835M variant (MAF = 0.0003) in our data, as less than a single minor allele would be expected within our entire sample of 979 participants.

*ENC1*, previously also known as *nuclear restricted protein/brain (NRP/B)*, is a gene that is highly expressed in developing neurons as well as in the adult neocortex and hippocampus in murine models. It colocalizes with actin [60] and is implicated in neurite development and neuronal process formation during neuronal differentiation [61]. In addition, *ENC1* is implicated in neural protection from various insults: *ENC1* is upregulated in vitro in settings of neural injury such as oxygen-glucose deprivation [62] or toxic intracellular protein aggregation and endoplasmic reticulum stress [63]. In these in vitro models, *ENC1* upregulation is shown to be detrimental for neural survival through its downregulation of cytoprotective genes such as *nuclear factor*, *erythroid 2 like 2 (NFE2L2*, also known as *NRF2)* [62] or its downregulation of the autophagic pathway through interaction with phosphorylated p62 [63]. In our study, rs76662990^G^ was not associated with baseline cognition, which makes it less likely that rs76662990^G^ results in significant neurodevelopmental differences. Moreover, rs76662990^G^ was associated with slower cognitive decline in multiple cognitive domains, which is consistent with widespread expression of *ENC1* in the mammalian neocortex [60]. Intriguingly, contrary to previous observations from in vitro models [62,63], we observed higher *ENC1* RNA level in participants with higher residual cognition, and higher *ENC1* RNA level was also correlated with less depressive symptoms, a known predictor of higher residual cognition [2]. Given the complexity of in vivo pathophysiology in the human brain and the vagaries of in vitro models, further study is required to elucidate the relationship between *ENC1* expression level and residual cognition.

In the *TMEM106B* locus, we found suggestive evidence of genetic association between better residual cognition and rs11509153^A^, which is in a strong LD with rs1990622^G^, a well-known protective allele against TDP-43 proteinopathy [37,55]. The *TMEM106B* haplotype captured by rs1990622 was first identified as a risk factor for FTLD with TDP-43 proteinopathy (FTLD-TDP) in a large GWAS [55], and later our group showed that the same haplotype is also implicated in TDP-43 proteinopathy burden in older adults without FTLD [37]. In the current study, our analyses show that rs11509153^A^ captures the same haplotype as the rs1990622^G^ allele, which is protective against FTLD-TDP. Moreover, epigenetic and transcriptomic associations with residual cognition observed at the *GRN* locus further suggest a role for the *TMEM106B–GRN* pathway in determining residual cognition. The association of the *TMEM106B* haplotype with residual cognition is plausible, as TDP-43 proteinopathy was not considered in our model to derive residual cognition. However, neither TDP-43 staging nor a semi-quantitative TDP-43 severity score fully explains the effect of rs11509153^A^ on residual cognition. Thus, it is possible that the *TMEM106B* haplotype is related to residual cognition via multiple different processes including processes independent of TDP-43 proteinopathy, which is in line with a prior study reporting that the *TMEM106B* genotype and TDP-43 proteinopathy have independent contributions to cognitive impairment in amyotrophic lateral sclerosis patients [64]. Nevertheless, it is also possible that current TDP-43 quantification methods are not adequately capturing the burden of TDP-43 proteinopathy.

With extensive data on each participant’s cognitive performance and detailed assessment of neuropathology, the ROS and MAP cohorts are uniquely positioned for studying the phenomenon of differential cognitive outcomes in the setting of neuropathology, as previously shown through multiple studies [1,2,4–7,9,14,15]. Nonetheless, our study has certain limitations. First, step 1 of our study was underpowered to detect genome-wide significance. To circumvent this problem, we leveraged our prior observation that independent genetic and epigenetic factors can converge on the same locus [19] and used a multistep approach with predefined significance thresholds to yield credible candidate genes. In step 1, we expected enrichment of pertinent genetic associations in the tail end of the *p*-value distribution, and, based on our experiences with previous GWASs in the ROS and MAP cohorts [24,25], we selected an arbitrary suggestive *p-*value threshold of 10^−5^ to detect a small number of loci that could be further tested in steps 2 and 3 without prohibitive multiple testing burden. Nonetheless, we may have missed pertinent genetic associations if SNPs from other loci either failed to reach our step 1 threshold or did not have coexisting epigenetic associations with residual cognition. Second, we selected genes only within 100 kb from the suggestive lead SNPs. This was to maximize signal-to-noise ratio based on the finding that most *cis*-regulatory elements are within 100 kb of TSSs [45]. Nonetheless, some regulatory elements can be more distant from the TSS [45], and we may have missed pertinent genes further than 100 kb from each SNP. However, interrogation of the GTEx database [48] and a previous brain eQTL meta-analysis [54] suggest that the loci identified in the step 1 analysis do not have regulatory roles for distant genes. Third, we used epigenetic and transcriptomic data from DLPFC alone, leaving the possibility that we may have missed genes that have significant roles in other brain regions or in other systems such as the immune system. Fourth, pathological phenotypes differed in the way they were quantified. For example, amyloid plaques and neurofibrillary tangles were counted and averaged throughout multiple brain regions, thus representing rigorous quantification, whereas other pathologies were recorded either as binary or categorical variables. The true effect of pathologies might have not been captured due to the variable definitions. Nonetheless, besides the *TMEM106B* locus—a risk factor for TDP-43 proteinopathy, which was not accounted for in our residual cognition model—none of the loci identified through the step 1 analysis were known genetic risk factors for the neuropathologies studied. Finally, given the limited sample size and power of our study, future replication studies as well as experimental studies are necessary to further clarify the role of the three genes identified through our study.

Despite its limitations, our study has initiated the genomic dissection of residual cognition, identifying three genes that deserve further investigation as determinants of differential cognitive outcomes in the setting of neuropathology. Further, this study supports our study design for addressing issues of limited statistical power in deeply phenotyped participants from cohorts of moderate size. As our results explain only a small portion of the dissociation between cognition and neuropathological burden, additional studies are required to elucidate the determinants of the unexplained cognitive variability in late life.

## Supporting information

S1 Table Characteristics of excluded participants. (DOCX)

S2 Table Cognitive tests shared in the Religious Orders Study and the Rush Memory and Aging Project. (DOCX)

S3 Table SNPs with suggestive associations with residual cognition (*p <* 1.0 × 10^−5^). (DOCX)

S4 Table Cognitive correlates of *UNC5C*, *ENC1*, and *TMEM106B* SNPs. (DOCX)

S5 Table Cognitive correlates of the loci not included in step 2 and 3. (DOCX)

## Abbreviations

AD: Alzheimer disease
CAA: cerebral amyloid angiopathy
*cis-*eQTL: *cis-*expression quantitative trait locus
DLPFC: dorsolateral prefrontal cortex
FDR: false discovery rate
FPKM: fragments per kilobase of transcript per million fragments mapped
FTLD: frontotemporal lobar degeneration
GTEx: Genotype-Tissue Expression
GWAS: genome-wide association study
LD: linkage disequilibrium
MAF: minor allele frequency
MAP: Rush Memory and Aging Project
QC: quality control
RNA-Seq: RNA sequencing
ROS: Religious Orders Study
SNP: single nucleotide polymorphism
TSS: transcription start site
