# Supplementary material for: Identification of genes associated with dissociation of cognitive performance and neuropathological burden: Multistep analysis of genetic, epigenetic, and transcriptional data
Source: PLoS Med. 2017 Apr 25;14(4):e1002287. doi: 10.1371/journal.pmed.1002287 (PMC5404753; doi:10.1371/journal.pmed.1002287)
Supplement: S1 Table — (DOCX) [file pmed.1002287.s001.docx]

**S1 Table. Characteristics of Excluded Participants**

|  | Included | Excluded | Total |
| --- | --- | --- | --- |
| Number of Participants, n | 979 | 418 | 1397 |
| Age at Enrollment, mean (SD) | 80.9 (6.9) | 80.9 (6.9) | 80.9 (6.9) |
| Age at Death, mean (SD) | 88.8 (6.4) | 86.8 (7.0) | 88.2 (6.6) |
| Female, n (%) | 630 (64.4%) | 274 (65.6%) | 904 (64.7%) |
| Education (years), mean (SD) | 16.4 (3.6) | 15.6 (4.0) | 16.1 (3.7) |
| Last Global Cognition, mean (SD) | -0.91 (1.19) | -0.88 (1.07) | -0.90 (1.15) |

Combined data from the Religious Orders Study and the Rush Memory and Aging Project are shown. Among 1,397 ROS-MAP participants who were deceased by the time of analysis (September 2014), 418 participants were excluded due to non-European ancestry or missing genotype, cognitive, or neuropathology data. Last Global Cognition= global cognitive performance proximate to death (z-score derived from baseline mean and standard deviation).
