# Supplementary material for: Identification of genes associated with dissociation of cognitive performance and neuropathological burden: Multistep analysis of genetic, epigenetic, and transcriptional data
Source: PLoS Med. 2017 Apr 25;14(4):e1002287. doi: 10.1371/journal.pmed.1002287 (PMC5404753; doi:10.1371/journal.pmed.1002287)
Supplement: S2 Table — (DOCX) [file pmed.1002287.s002.docx]

**S2 Table. Cognitive Tests Shared in the Religious Orders Study and the Rush Memory and Aging Project**

| Test | Composite |
| --- | --- |
| Logical Memory Ia | Episodic memory |
| Logical Memory IIa | Episodic memory |
| Immediate Story Recall | Episodic memory |
| Delayed Story Recall | Episodic memory |
| Word List Memory (3 trials) | Episodic memory |
| Word List Recall | Episodic memory |
| Word List Recognition | Episodic memory |
| Boston Naming Test | Semantic memory |
| Category Fluency (fruits, animals) | Semantic memory |
| National Adult Reading Test | Semantic memory |
| Digit Span Forward | Working memory |
| Digit Span Backward | Working memory |
| Digit Ordering | Working memory |
| Symbol Digit Modalities Test | Perceptual speed |
| Number Comparison | Perceptual speed |
| Judgment of Line Orientation | Visuospatial ability |
| Standard Progressive Matrices | Visuospatial ability |
