# Supplementary material for: Identification of genes associated with dissociation of cognitive performance and neuropathological burden: Multistep analysis of genetic, epigenetic, and transcriptional data
Source: PLoS Med. 2017 Apr 25;14(4):e1002287. doi: 10.1371/journal.pmed.1002287 (PMC5404753; doi:10.1371/journal.pmed.1002287)
Supplement: S4 Table — (DOCX) [file pmed.1002287.s004.docx]

**S4 Table. Cognitive Correlates of *UNC5C,* *ENC1*, and *TMEM106B* SNPs**

Estimated effect indicates change in each cognitive score, as measured by z-score, per each additional minor allele of each SNP.

FDR, false discovery rate; SNP, single nucleotide polymorphism
